# Low‐Value Prescribing Among Older Adults With Psychiatric Disorders in a Lebanese Geriatric Care Hospital: A Cross‐Sectional Study

**DOI:** 10.1002/hsr2.72799

**Published:** 2026-07-12

**Authors:** Bahia Chahine, Fatima Hassane, Haya Hamed, Hana El Zohbi, Rania El Majzoub

**Affiliations:** ^1^ School of Pharmacy Lebanese International University Beirut Lebanon

**Keywords:** EVOLV‐Rx, Lebanon, low value prescription, older adults, polypharmacy, psychiatric disorders

## Abstract

**Background and Aims:**

Low‐value prescribing (LVP) refers to the use of medications whose potential risks or costs outweigh their expected clinical benefits and is increasingly recognized as an important concern in the pharmacological management of older adults. This study aimed to assess the prevalence of low‐value prescribing (LVP) and associated factors among hospitalized older adults with psychiatric disorders in Lebanon using the Evaluating Opportunities to Decrease Low‐Value Prescribing‐Rx (EVOLV‐RX) tool.

**Methods:**

A cross‐sectional study was conducted from May 2022 to December 2022 among hospitalized patients aged ≥ 60 years with diagnosed psychiatric disorders receiving care in the geriatric division of a care hospital in Beirut during the study period. Patients with complete medical records and receiving at least one chronic medication were eligible for inclusion. Low‐value prescribing was assessed using the EVOLV‐Rx tool. Descriptive statistics were used to describe independent variables. Bivariate and logistic regression analyses were performed to identify factors associated with LVP.

**Results:**

The study included 180 patients, 128 (71.1%) of whom had at least one low‐value prescription. The most frequently identified LVP was the use of proton pump inhibitors for more than two consecutive months, observed in 57 patients (31.7%), followed by the concomitant use of highly anticholinergic drugs (47, 26.1%), therapeutic duplication of antipsychotics and sleep medications (25, 13.9%), and therapeutic duplication of antipsychotics (22, 12.2%). In adjusted analyses, both polypharmacy and smoking were significantly associated with the presence of low‐value prescribing.

**Conclusion:**

LVP was highly prevalent among hospitalized older adults in a psychogeriatric care setting in Lebanon. The observed associations with polypharmacy and smoking highlight the importance of structured medication review and deprescribing strategies in this population.

## 1 Background

The United Nations defines older adults as those who are over 60 years old [1]. This population represents one of the fastest‐growing demographic groups worldwide. According to United Nations population projections, the proportion of individuals aged 60 years and older is expected to reach approximately 22% of the global population by 2050 [2].

Low‐value prescribing (LVP) is defined as the use of medications whose costs or risks outweigh their potential benefits [3]. It is commonly observed in the context of inappropriate medication use and polypharmacy (the use of ≥ 5 medications), both of which are prevalent among older adults [3].

This highlights a related but conceptually distinct issue known as potentially inappropriate medications (PIMs). PIMs are drugs that, when administered to older adults, carry a higher risk of adverse drug events (ADEs), defined as patient harm resulting from medication use, than their expected clinical benefits [4]. While PIMs primarily reflect medication safety concerns affecting individual patients, low‐value prescribing represents a broader challenge that also affects the efficiency and sustainability of healthcare systems [5].

Understanding low‐value prescribing may therefore help payers and health systems design interventions that better mitigate medication‐related risks and reduce unnecessary medication use associated with polypharmacy, although the economic impact of these practices was not directly evaluated in the present study [6]. In particular, identifying low‐value prescribing may support deprescribing strategies to reduce medication burden and improve medication safety among older adults.

Several instruments have been developed to improve prescribing quality, including physician‐led guidelines and tools such as the Beers criteria and the Screening Tool of Older Persons' Prescriptions (STOPP) criteria, which primarily focus on identifying potentially inappropriate prescribing practices [7, 8]. However, these instruments typically target specific populations, such as older adults [9]. Recent campaigns have led to the development of nationwide lists of low‐value procedures that are specialty‐specific and not restricted to any particular subpopulation [10]. Certain initiatives developed programs such as the UK's National Institute for Health and Care Excellence (NICE) and the Choosing Wisely campaign, which promote evidence‐based recommendations to discourage low‐value interventions. Other projects of a similar nature are the EVOLVE program of the Royal Australasian College of Physicians, the Too Much Medicine series of the British Medical Journal, and the Less is More series of the Journal of the American Medical Association [11].

While PIMs are addressed by Beers and STOPP criteria, potential prescribing omissions (PPOs) are covered by the Screening Tool to Alert to Right Treatment (START) criteria [12]. Evaluating Opportunities to Decrease Low‐Value Prescribing‐Rx (EVOLV‐Rx) is a value‐based clinical metric designed to identify and quantify low‐value prescribing practices in older adults by synthesizing evidence‐based recommendations that extend beyond medication safety alone. It stands apart from other metrics by providing a methodical way to define, rank, and quantify LVP practices that can be operationalized in payer and provider data [13].

This distinction makes EVOLV‐Rx particularly relevant in contexts such as Lebanon, where previous studies have primarily focused on PIMs using the Beers and STOPP criteria, leaving a gap in the evaluation of low‐value prescribing from a broader, value‐based perspective. In addition, EVOLV‐Rx may lessen polypharmacy, improve the identification of LVPs in conjunction with other low‐value tests and treatments, and allow geriatrics to obtain high‐value care throughout the whole range of healthcare services in a manner that is consistent with their beliefs and perspectives [13].

Recent evidence suggests that inappropriate prescribing remains a common issue in Lebanon. A study conducted in 2018 assessed PIM in older adults according to Beers and STOPP criteria, which showed that 60% of the patients had at least one potentially inappropriate medication [14]. Another study reported that 23.9%–61.8% of older adults in Lebanon were exposed to polypharmacy [15]. These findings highlight the need for more comprehensive evaluation tools, which justifies the use of EVOLV‐Rx in this study to capture low‐value prescribing practices beyond traditional PIM assessment. Therefore, this study aimed to assess the prevalence and factors associated with low‐value prescribing among patients aged 60 years and older treated at an elderly care hospital, using the EVOLV‐Rx tool.

## 2 Methods

### 2.1 Study Setting

A cross‐sectional study was conducted in the geriatric division of an elderly care hospital in Beirut, Lebanon, from May 2022 to December 2022. The hospital includes a geriatrics division that provides healthcare for about 300 resident‐patients (long‐stay inpatients) with multiple chronic conditions, life‐limiting illness, frailty, or disability, and a psychogeriatric division that provides care for 300 resident‐patients with psychiatric disorders.

### 2.2 Participants, Recruitment, and Ethics

The study sample included hospitalized patients aged ≥ 60 years with diagnosed psychiatric illness who received a comprehensive geriatric assessment by a trained physician during the study period and were taking at least one chronic medication. Chronic medication was defined as any medication prescribed for continuous use for at least 3 months before data collection. Exclusion criteria included patients younger than 60 years. All patients who met the inclusion criteria during the study period were identified through a review of hospital census records and screened for eligibility. Data were collected for all eligible patients. Prescription data and clinical variables were manually extracted from hard‐copy medical records by the principal investigator using a standardized data‐collection form. To ensure accuracy, extracted data were systematically reviewed for completeness before statistical analysis. The form included demographic characteristics (i.e., age, gender, weight, height, marital status, nationality, and education), duration of hospital stay, medical history, number of medications, their frequency, and their reported side effects. Functional status and dependency were assessed using the Katz ADL Scale.

The data in this study have been collected and analyzed in compliance with privacy and data protection regulations. In total, 180 patients met the inclusion criteria, had complete medical records, and were included in the final analysis; no additional exclusions were applied beyond the predefined criteria. No written informed consent was required from patients, as this study posed no physiological, psychological, or social risks given its observational nature. The ethics committee approved a waiver of written informed consent due to the retrospective observational design. All extracted data were fully anonymized before analysis. This study was approved by the ethics committee of Lebanese International University School of Pharmacy (2022RC‐014‐LIUSOP) and adheres to the Declaration of Helsinki.

### 2.3 Measures

Low‐value prescribing was assessed using the EVOLV‐Rx tool, a validated value‐based metric that identifies and quantifies low‐value prescribing practices in older adults. EVOLV‐Rx applies predefined criteria based on patient characteristics, including age, clinical history, and medication profiles [13]. The EVOLV‐Rx framework was developed through two steps: step 1: Generate criteria to detect candidate low‐value prescribing practices; step 2: Convene an online modified Delphi panel [13].

For each patient, the complete medication list was reviewed and mapped against the validated EVOLV‐Rx criteria. Each medication was evaluated according to patient age, diagnosis, duration of therapy, and relevant prescribing context. When a medication met predefined EVOLV‐Rx criteria for low‐value prescribing, it was assigned the appropriate classification. Patients were categorized as having low‐value prescribing if at least one criterion was met [13].

All EVOLV‐Rx assessments were performed by a trained clinical pharmacist using standardized criteria to ensure consistent classification.

To minimize subjectivity, EVOLV‐Rx criteria were applied with predefined operational definitions derived from the original framework, ensuring standardized classification across all patients. The complete mapping of medications under each LVP category is available in Supporting Information S1: Table S1.

In the original development study by Radomski et al., 27 candidate low‐value prescribing practices were identified, of which 16–18 were ultimately validated and included in the final EVOLV‐Rx framework [13]. In the present study, the medication‐specific criteria provided in the EVOLV‐Rx supporting material were applied, resulting in 26 operational low‐value prescribing items grouped under seven conceptual domains. These items represent medication‐level indicators derived from the original EVOLV‐Rx framework and were used to identify low‐value prescribing practices in the study population while maintaining conceptual consistency with the validated tool. Functional status was assessed using the Katz Index of Independence in Activities of Daily Living (ADL), which measures the patient's ability to perform ADLs independently. Katz's score of 6 indicates full function, 4 indicates moderate impairment, and 2 or less indicates severe functional impairment [16]. The Charlson comorbidity index (CCI) was also used in this study; it predicts the risk of 1‐year mortality after hospitalization among patients with specific comorbid conditions. It includes 19 conditions [17]. It was categorized as mild (0–1), moderate (2–3), and severe (≥ 4) for descriptive and analytical purposes.

### 2.4 Data Analysis

Statistical analyses were performed using IBM SPSS Statistics for Windows, version 26. Continuous variables were represented as means and standard deviations, while categorical variables were presented as frequencies and percentages. The independent‐samples *t*‐test was performed for continuous variables, and the chi‐square test was performed for categorical variables. No a priori sample size calculation or formal power analysis was performed because this study included all eligible patients who met the inclusion criteria during the study period. Therefore, the final sample size reflects the total available eligible population rather than a pre‐specified target sample size. This should be considered when interpreting nonsignificant findings, as the study may have been underpowered to detect weaker associations. The dependent variable was coded as the presence of at least one low‐value prescription, per the EVOLV‐Rx tool (LVP = 1), versus no low‐value prescription (LVP = 0). Polypharmacy was included as a binary independent variable, defined as the concurrent use of 5 or more medications. Patients receiving fewer than five medications (< 5) served as the reference category. Smoking status was included because it may reflect differences in comorbidity burden, health behaviors, and medication exposure, which could influence prescribing patterns in older adults. It was also entered as a binary variable, with non‐smokers as the reference group.

Binary logistic regression was used to identify the independent variables that remained associated with low‐value prescriptions while controlling for potential confounders. Because of the limited sample size (*n* = 180), variables with *p* values < 0.20 in the bivariate analysis were entered into the multivariate logistic regression model to avoid overfitting while retaining potentially meaningful predictors. Clinically relevant variables identified in prior literature were also considered during model construction to control for potential confounding. This approach is commonly used in small‐sample studies to achieve a reliable model fit. Adjusted odds ratios (aORs) with 95% confidence intervals (CIs) were reported. All statistical tests were two‐sided, and *p *< 0.05 was considered statistically significant.

## 3 Results

In this cross‐sectional study, 180 patients met the inclusion criteria and were included in the final analysis. All participants were aged ≥ 60 years, had complete medical records, and received at least one chronic medication during the study period.

The study population included 95/180 females (52.8%), with a mean age of 75 ± 9.2 years. Patients were hospitalized for 127 ± 141 days with a median of 83 days (IQR: 25–170) and were prescribed 6.4 ± 3.4 medications (median 6 drugs, IQR 4–9 medications, range from 1 to 16 medications) during their hospital stay. Polypharmacy was present in more than two‐thirds of patients (121/180 patients (67.2%). Based on the Charlson Comorbidity Index (CCI), 76/180 patients (42.2%) were classified as having severe comorbidity. According to the Katz ADL score, 96/180 patients (53.3%) were classified as dependent. Overall, 128/180 patients (71.1%) had at least one low‐value prescription identified using the EVOLV‐Rx tool.

The demographics and physiological characteristics of the study population (*n *= 180) are presented in Table 1.

**Table 1:** Demographic and clinical characteristics of participants.

Characteristics	Number of subjects (%)
Age in years (mean ± SD)	75.49 ± 9.2
Gender	
Male	85 (47.2)
Female	95 (52.8)
Marital status	
Single	88 (48.9)
Married	92 (51.1)
Education	
No schooling	78 (43.3)
Schooling	102 (56.7)
Living	
Alone	48 (26.7)
With family	132 (73.3)
Smoking	
Smoker	69 (38.3)
Non‐smoker	111 (61.7)
Alcohol	
Alcoholic	5 (2.8)
Non‐alcoholic	175 (97.2)
Insurance	
Yes	25 (13.9)
No	155 (86.1)
Length of hospital stay (days), median (IQR)	83 (25–170)
Charlson Comorbidity Index	
Mild (1–2)	34 (18.9)
Moderate (3–4)	70 (38.9)
Severe (≧ 5)	76 (42.2)
KATZ ADL score	
Dependent (0–2)	96 (53.3)
Partially dependent (3–5)	21 (11.7)
Independent (6)	63 (35)
Polypharmacy (=> 5)	121 (67.2)
Low‐value prescription	128 (71.1)
Psychiatric illnesses	
Schizophrenia	83 (46.1)
Mental retardation	29 (16.1)

*Note:* LVP: low‐value prescribing defined as the presence of ≥ 1 low‐value prescription according to EVOLV‐Rx criteria.

**Figure 1:**
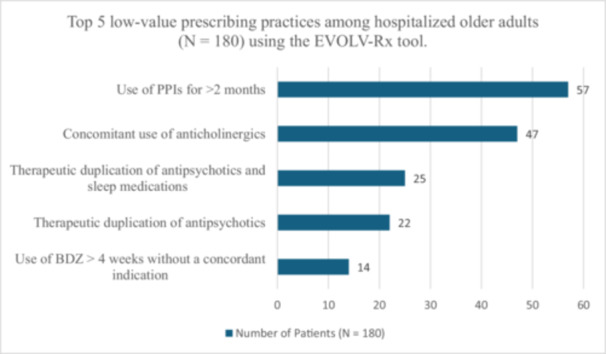
Top five most frequent low‐value prescribing practices among hospitalized older adults (*N* = 180), identified using the EVOLV‐Rx tool. Values represent the number of patients (*n*) and the percentage (%). Abbreviations: BDZ, benzodiazepines; PPI, proton pump inhibitors.

Table 2 summarizes the distribution of low‐value prescribing across EVOLV‐Rx categories. Within the potentially unsafe use category, the most frequent items were concomitant use of highly anticholinergic drugs (47/180 patients, 26.1%) and therapeutic duplication of antipsychotics and sleep medications (25/180 patients, 13.9%). Within the prolonged use category, prolonged proton pump inhibitor (PPI) use for more than 2 months was the most frequent pattern (57/180 patients, 31.7%). Under the inappropriate medication use, therapeutic duplication of antipsychotics (22/180 patients, 12.2%) and prolonged antipsychotic use in dementia without serious mental illness (11/180 patients, 6.1%) were most common. The uncertain clinical usefulness category was less frequent, with baby aspirin use in adults aged ≥ 70 years without ASCVD risk observed in 3/180 patients (1.7%). Figure 1 presents the top five most frequent low‐value prescribing practices identified among hospitalized older adults (*N *= 180) using the EVOLV‐Rx tool. A complete list of medications identified under each EVOLV‐Rx low‐value prescription (LVP) item is provided in Supporting Information S1: Table S1.

**Table 2:** Frequency of low‐value prescribing practices by EVOLV‐Rx category (*N* = 180).

Criteria	Number of subjects (%)
1. Potentially unsafe use	
1. Concomitant use of highly anticholinergic drugs	47 (26.1)
2. Use of DAPT for > 6 months in patients at increased risk of bleeding	3 (1.7)
3. Therapeutic duplication of BDZ and anticonvulsants	3 (1.7)
4. Use of BDZ > 4 weeks without a concordant indication	14 (7.8)
5. Therapeutic duplication of BDZ and antipsychotics	4 (2.2)
6. Therapeutic duplication of antipsychotics and sleep medications	25 (13.9)
7. Therapeutic duplication of antipsychotics and anticonvulsants	5 (2.8)
8. Therapeutic duplication of antipsychotics and skeletal muscle relaxants	3 (1.7)
9. Therapeutic duplication of antipsychotics and skeletal muscle relaxants	2 (1.1)
10. Concomitant use of antidepressant and BDZ	3 (1.7)
2. Prolonged use	
1. PPI use for more than 2 consecutive months	57 (31.7)
2. NSAID use for more than 90 consecutive days	2 (1.1)
3. Uncertain clinical usefulness	
1. Use of baby aspirin in adults aged 70 and above without evidence of ASCVD	3 (1.7)
2. Use of sleep medication for a total of more than 4 weeks	1 (0.6)
4. Ineffective use	
1. Use of gabapentinoids without diagnosis of post‐herpetic neuralgia or neuropathic pain	2 (1.1)
2. Use of thyroid hormone in more than 80 years old	1 (0.6)
5. Inappropriate use	
1. Use of antipsychotics for more than 90 days in patients with dementia without evidence of underlying serious mental illness	11 (6.1)
2. Use of vitamin B12 without an appropriate diagnosis of vitamin B12 deficiency or anemia	2 (1.1)
3. Use of ACHE‐ to treat severe or end‐stage Alzheimer's disease	5 (2.8)
4. Therapeutic duplication of antipsychotics	22 (12.2)
5. Concurrent use of antiparkinsonian medication and antipsychotic medication	2 (1.1)
6. Uncertain scientific validity	
1. Use of iron without an appropriate diagnosis of anemia	3 (1.7)
2. Use of statins in adults without a diagnosis of atherosclerotic cardiovascular disease	1 (0.6)
3. Use of antihypertensive medication class that is poorly tolerated in older adults	6 (3.3)
4. Concomitant use of loop diuretics and DHP‐CCB	7 (3.9)
7. Overly intensive treatment	
1. Use of high‐risk antidiabetic agents	5 (2.8)

Abbreviations: ACHE‐I, acetylcholinesterase inhibitors; ASCVD, atherosclerotic cardiovascular disease; BDZ, benzodiazepines; DAPT, dual antiplatelet therapy; DHP‐CCB, dihydropyridine calcium channel blockers; NSAID, nonsteroidal anti‐inflammatory drug; PPI, proton pump inhibitors.

Definitions:

Potentially unsafe use: medications associated with increased risk of adverse drug events in older adults.

Prolonged use: medications used beyond the recommended duration.

Uncertain clinical usefulness: medications with limited evidence of benefit.

Ineffective use: medications prescribed without an appropriate indication.

Uncertain scientific validity: practices lacking sufficient supporting evidence.

Overly intensive treatment: treatments exceeding recommended targets.

As shown in Table 3, bivariate analyses showed no significant associations between low‐value prescribing and sociodemographic characteristics, Katz ADL score, or CCI. However, statistically significant associations were observed between low‐value prescribing and polypharmacy (*p *< 0.001) and smoking status (*p *= 0.01). In the multivariable logistic regression analysis, both polypharmacy and smoking remained independently associated with the presence of low‐value prescribing. Patients with polypharmacy had significantly higher odds of having at least one low‐value prescription compared with those without polypharmacy (aOR = 6.59, 95% CI: 3.18–13.69; *p *< 0.001).

**Table 3:** Bivariate and logistic regression analysis to identify factors associated with low‐value prescription (EVOLV‐RX).

Variable	Bivariate analysis			**Regression analysis**	
	**Low value prescription (EVOLV‐RX) = 0 (*n* = 52), (*n*, %)**	**Low value prescription (EVOLV‐RX) = 1 (*n* = 128), (*n*, %)**	*p* value	**aOR (95% CI)**	*p* value
Age			0.260		
Gender			0.40		
Male	22 (42.3)	63 (47.2)			
Female	30 (57.7)	65 (52.8)			
Marital status			0.89		
Single	25 (48.1)	63 (49.2)			
Married	27 (51.9)	65 (50.8)			
Smoking			0.01	2.80 (1.27–6.16)	0.011
Non‐smoker	39 (75)	72 (56.3)			
Smoker	13 (25)	56 (43.8)			
Alcohol			0.14		
Non‐alcoholic	52 (100)	123 (96.1)			
Alcoholic	0 (0)	5 (3.9)			
Education			0.87		
Not‐educated	23 (44.2)	55 (43)			
Educated	29 (55.8)	73 (57)			
Insurance			0.29		
No	47 (90.4)	108 (84.4)			
Yes	5 (9.6)	20 (15.6)			
Polypharmacy			< 0.001	6.59 (3.18–13.69)	< 0.001
No	32 (61.5)	27 (21.1)			
Yes	20 (38.5)	101 (78.9)			
KATZ ADL score			0.80		
Dependent (0–2)	29 (55.8)	96 (53.3)			
Partially dependent (3–5)	5 (9.6)	21 (11.7)			
Non‐dependent (6)	18 (34.6)	63 (35)			
Charlson comorbidity index			0.87		
Mild	11 (21.2)	23 (18)			
Moderate	20 (38.5)	50 (39.1)			
Severe	21 (40.4)	55 (43)			

*Note:* Outcome variable: LVP (1 = presence of ≥ 1 low‐value prescription; 0 = none).

Reference categories: no polypharmacy (< 5 medications), non‐smoker, male sex, not educated, no insurance, dependent (Katz ADL), and mild CCI.

CCI categories were defined as mild (0–1), moderate (2–3), and severe (≥ 4).

*p* values in bivariate analysis were derived using *χ* ^2^ tests for categorical variables and independent samples *t*‐tests for continuous variables.

Abbreviations: aOR, adjusted odds ratio; CI, confidence interval; CCI, Charlson Comorbidity index; Katz ADL, Katz index of independence in activities of daily living; LVP, low‐value prescribing.

Similarly, smoking was independently associated with increased odds of low‐value prescribing (aOR = 2.80, 95% CI: 1.27–6.16; *p *= 0.011). On the other hand, other variables, including CCI and Katz ADL score, were not statistically associated with low‐value prescribing.

## 4 Discussion

To the best of our knowledge, this is the first study in Lebanon to apply the EVOLV‐Rx metric to evaluate low‐value prescribing in older adults. It is a recently developed tool that may help physicians and pharmacists identify low‐value prescribing practices relevant to deprescribing in older adults.

A high prevalence of low‐value prescribing (71.1%) was observed in this psychogeriatric inpatient population. Reported prevalence estimates vary across studies depending on study design and patient population. For example, one longitudinal study reported that more than half of participants experienced at least one episode of low‐value prescribing, while 16% had persistent exposure over 24 months [18]. Variations may influence differences in the prevalence of low‐value prescribing across studies in study design, patient populations, and clinical settings. In particular, differences in inclusion criteria and case mix, such as the present study's focus on long‐term hospitalized patients with psychiatric comorbidities, compared with dementia‐specific patients in other studies, may contribute to variability in reported prevalence [18]. Additional methodological and health system factors, including differences in prescribing guidelines and healthcare organizations, may also contribute to variability between studies [19].

Low‐value prescribing was strongly associated with polypharmacy in the present study (aOR = 6.59; 95% CI: 3.18–13.69) when using the EVOLV‐Rx metric. Higher medication burden and the presence of potentially inappropriate medications were associated with a greater likelihood of low‐value prescribing. However, polypharmacy should be interpreted primarily as a marker of overall medication burden and prescribing complexity, rather than a causal determinant of low‐value prescribing, because a higher number of medications increases the probability that at least one low‐value prescription is present. Similar associations between polypharmacy and inappropriate prescribing patterns have been reported in a longitudinal study [18]. In addition, several studies have shown that community‐dwelling older adults exposed to polypharmacy are at greater risk of adverse drug events, hospitalizations, and LVP [20, 21, 22]. Given the cross‐sectional design of the study, the temporal direction of this relationship cannot be established, and polypharmacy and low‐value prescribing should be interpreted as co‐occurring phenomena rather than causally linked.

With respect to polypharmacy, the prevalence of a longitudinal study was more than our study by 15% [18]. This difference may reflect variations in study populations, clinical settings, and inclusion criteria rather than true differences in prescribing behavior [18, 23]. On the other hand, another study conducted in Poland reported that 53.5% of older adults aged ≥ 65 years were exposed to polypharmacy [24], which is less than our study by 13%. Differences in age thresholds and study design may also partially explain the variation in reported polypharmacy prevalence across studies.

In the presence of low‐value prescribing, multimorbidity, and polypharmacy, the complexity of caring for older adults highlights the need for deprescribing, particularly among patients with multiple comorbidities. Therefore, low‐value prescribing represents an important challenge in optimizing medication use and ensuring appropriate pharmacotherapy in older adults. Moreover, the EVOLV‐Rx tool may support structured medication review processes and guide clinicians in identifying potentially low‐value therapies during chronic medication management [25]. These findings add to the growing body of evidence on low‐value prescribing in older adults and may inform future interventions aimed at medication optimization and deprescribing among patients with multimorbidity.

Smoking was included in the analysis as a potential marker of clinical and behavioral complexity and was independently associated with low‐value prescribing in the adjusted model (aOR = 2.80, 95% CI: 1.27–6.16). This association should be interpreted with caution, as the cross‐sectional design does not permit causal inference. In this population, smoking may reflect a broader profile of multimorbidity and greater exposure to psychotropic and chronic medications, which may increase the complexity of prescribing decisions and the likelihood of low‐value prescribing. Smoking status may therefore serve as a marker of underlying clinical complexity not fully captured by the variables included in the present analysis.

Despite the limited number of statistically significant predictors, the high prevalence of low‐value prescribing observed in this study remains clinically important. These findings highlight the potential value of tools such as EVOLV‐Rx in supporting medication assessment and identifying opportunities to improve prescribing practices among older adults. Several limitations in this study could be addressed in future studies. First, the study population consisted of hospitalized older adults with psychiatric disorders who were receiving chronic medications in a psychogeriatric care setting. Therefore, the findings may not be generalizable to community‐dwelling older adults or to older adults without psychiatric illness. Second, the study was conducted at a single center; therefore, multicenter studies that include more diverse geriatric populations are needed to improve the generalizability of the findings. Moreover, the limited number of prior EVOLV‐Rx studies restricts direct comparison of findings. In addition, no a priori sample size calculation was performed, as all eligible patients during the study period were included. Therefore, the relatively small sample size (*n* = 180) may have limited the statistical power of the regression analysis, reduced the precision of the estimates, and increased the risk of type II error, particularly for weaker associations. The influence of chance in variable selection should also be considered, since only variables with *p* values < 0.20 in bivariate analysis were included in the final model. Accordingly, the limited number of statistically significant predictors observed in the adjusted analysis should be interpreted cautiously. Future studies with larger samples and a more balanced representation of comorbidities should be multicenter, incorporate covariates based on clinical relevance and prior evidence to strengthen the model's generalizability and robustness, and place greater emphasis on the Charlson Comorbidity Index (CCI) and the Katz ADL score.

## 5 Conclusion

This study showed that low‐value prescribing, as identified using the EVOLV‐Rx tool, was highly prevalent among hospitalized older adults with psychiatric disorders in a psychogeriatric care setting and was associated with smoking and polypharmacy. These findings highlight the potential role of clinical pharmacists in detecting low‐value prescriptions. Potential pharmacist‐led interventions include systematic medication review using EVOLV‐Rx criteria, identification and prioritization of potentially low‐value therapies, collaboration with prescribers to support deprescribing decisions, and ongoing monitoring to optimize medication regimens and reduce unnecessary treatment burden. Future studies should include more diverse geriatric populations, employ multicenter designs, and place greater emphasis on the Charlson Comorbidity Index (CCI) and the Activities of Daily Living (ADL) scale.

## Author Contributions

**Bahia Chahine:** conceptualization, methodology, writing – review and editing, writing – original draft, formal analysis. **Fatima Hassane:** investigation, methodology, writing – original draft. **Haya Hamed:** writing – review and editing. **Hana El Zohbi:** writing – review and editing, formal analysis. **Rania El Majzoub:** writing – review and editing.

## Funding

The authors have nothing to report.

## Ethics Statement

This study was approved by the Institutional Review Board at the Lebanese International University (approved number 2022RC‐014‐LIUSOP).

## Conflicts of Interest

The authors have no conflicts of interest.

## Transparency Statement

Bahia Chahine affirms that this manuscript is an honest, accurate, and transparent account of the study being reported, that no important aspects of the study have been omitted, and that any discrepancies from the study as planned (and, if relevant, registered) have been explained.

## Supporting information


Supporting File

## Data Availability

The data that support the findings of this study are available on request from the corresponding author.
