# Supplementary material for: Low‐Value Prescribing Among Older Adults With Psychiatric Disorders in a Lebanese Geriatric Care Hospital: A Cross‐Sectional Study
Source: Health Sci Rep. 2026 Jul 12;9(7):e72799. doi: 10.1002/hsr2.72799 (PMC13358215; doi:10.1002/hsr2.72799)
Supplement: Supplementary file 1 — Supporting File [file HSR2-9-e72799-s001.docx]

**Table S1:** ***List of the medications identified under each of the 26 low-value prescription (LVP) categories assessed using the EVOLV-Rx tool.***

| **LVP Category** | **LVP Item** | **Medications** |
| --- | --- | --- |
| **1. Potentially Unsafe Use** | Concomitant use of highly anticholinergic drugs | Promethazine, Trihexyphenidyl, Clomipramine, Chlorpromazine, Clozapine, Chlorpheniramine |
|  | Use of DAPT for > 6 months in patients at increased risk of bleeding | Aspirin + clopidogrel |
|  | Therapeutic duplication of BDZ & anticonvulsants | Lorazepam, Diazepam, Clonazepam + Valproate, Carbamazepine |
|  | Use of BDZ for> 4 weeks without a concordant indication | Lorazepam, Diazepam, Clonazepam, Alprazolam |
|  | Therapeutic duplication of BDZ & antipsychotics | Lorazepam, Diazepam, Alprazolam + Clozapine, Risperidone, Haloperidol, Chlorpromazine |
|  | Therapeutic duplication of antipsychotics & sleep medications | Clozapine, Quetiapine, Risperidone, Haloperidol, Chlorpromazine, Olanzapine + Zolpidem, Promethazine |
|  | Therapeutic duplication of antipsychotics & anticonvulsants | Clozapine, Quetiapine, Chlorpromazine, Zuclopenthixol + Carbamazepine, Valproate, Levetiracetam |
|  | Therapeutic duplication of antipsychotics & skeletal muscle relaxants | Clozapine, Quetiapine, Haloperidol, Chlorpromazine + Tizanidine, Trihexyphenidyl |
|  | Therapeutic duplication of skeletal muscle relaxants & anticonvulsants | Tizanidine, Trihexyphenidyl + Valproate, Carbamazepine, Levetiracetam |
|  | Concomitant use of antidepressant and BDZ | Clomipramine, Escitalopram + Lorazepam, Diazepam, Alprazolam |
| **2. Prolonged Use** | PPI use for more than two consecutive months | Omeprazole, Esomeprazole, Rabeprazole, Lansoprazole |
|  | NSAID use for more than 90 consecutive days | Ibuprofen |
| **3. Uncertain Clinical Usefulness** | Use of baby aspirin in adults ≥70 without ASCVD | Aspirin |
|  | Use of sleep medication for >4 weeks | Zolpidem, Eszopiclone, Diazepam |
| **4. Ineffective Use** | Use of gabapentinoids without diagnosis of post-herpetic neuralgia or neuropathic pain | Gabapentin, Pregabalin |
|  | Use of thyroid hormone in adults >80 | Levothyroxine |
| **5. Inappropriate Use** | Use of antipsychotics >90 days in dementia without serious medical illness | Clozapine, Risperidone, Olanzapine, Quetiapine, Haloperidol |
|  | Use of vitamin B12 without deficiency or anemia | Vitamin B12 |
|  | Use of AChE inhibitors in severe/end-stage Alzheimer’s | Donepezil, Rivastigmine |
|  | Therapeutic duplication of antipsychotics | Clozapine, Risperidone, Haloperidol, Quetiapine, Chlorpromazine, Zuclopenthixol |
|  | Concurrent use of antiparkinsonian & antipsychotic meds | Trihexyphenidyl, (Levodopa+Carbidopa) + Clozapine, Risperidone, Chlorpromazine |
| **6. Uncertain Scientific Validity** | Use of iron without diagnosis of anemia | Iron supplements |
|  | Use of statins in adults without ASCVD | Atorvastatin |
|  | Use of antihypertensive class poorly tolerated in older adults | Propranolol, Diltiazem, Tamsulosin |
|  | Concomitant use of loop diuretic & DHP-CCB | Furosemide + Amlodipine |
| **7. Overly Intensive Treatment** | Use of high-risk antidiabetic agents | Insulin, Gliclazide |
